# Supplementary material for: Design and 3D Printing of Stretchable Conductor with High Dynamic Stability
Source: Materials (Basel). 2023 Apr 14;16(8):3098. doi: 10.3390/ma16083098 (PMC10146708; doi:10.3390/ma16083098)
Supplement: Supplementary file 1 [file materials-16-03098-s001.zip › materials-2312850-supplementary.pdf]

Supporting Information

Design and 3D Printing of Stretchable Conductor with High Dynamic Stability

Chao Liu [1,2,3], Yuwei Wang [2,3], Shengding Wang [2,3], Xiangling Xia [2,3], Huiyun Xiao [2,3], Jinyun Liu [2,3], Siqi Hu [2,3], Xiaohui Yi [2,3], Yiwei Liu [2,3], Yuanzhao Wu [2,3], Jie Shang [2,3,*] and Run-Wei Li [4,*]

[1] School of Materials Science and Chemical Engineering, Ningbo University, Ningbo 315211, China
[2] CAS Key Laboratory of Magnetic Materials and Devices, Ningbo Institute of Materials Technology and Engineering, Chinese Academy of Sciences, Ningbo 315201, China
[3] Zhejiang Province Key Laboratory of Magnetic Materials and Application Technology, Ningbo Institute of Materials Technology and Engineering, Chinese Academy of Sciences, Ningbo 315201, China
[4] College of Materials Science and Opto-Electronic Technology, University of Chinese Academy of Sciences, Beijing 100049, China
* Correspondence: shangjie@nimte.ac.cn (J.S.); runweili@nimte.ac.cn (R.-W.L.)

Keywords: liquid metal; stretchable conductor; 3D printing; high dynamic stability; wearable devices

Table S1. Factors and levels of orthogonal experiment

Control Factors	Level 1	Level 2	Level 3
Shape	1	2	3
External diameter (mm)	3.0	4.0	5.0
Internal diameter (mm)	0.5	1.0	1.5
Length (mm)	3.0	4.0	5.0

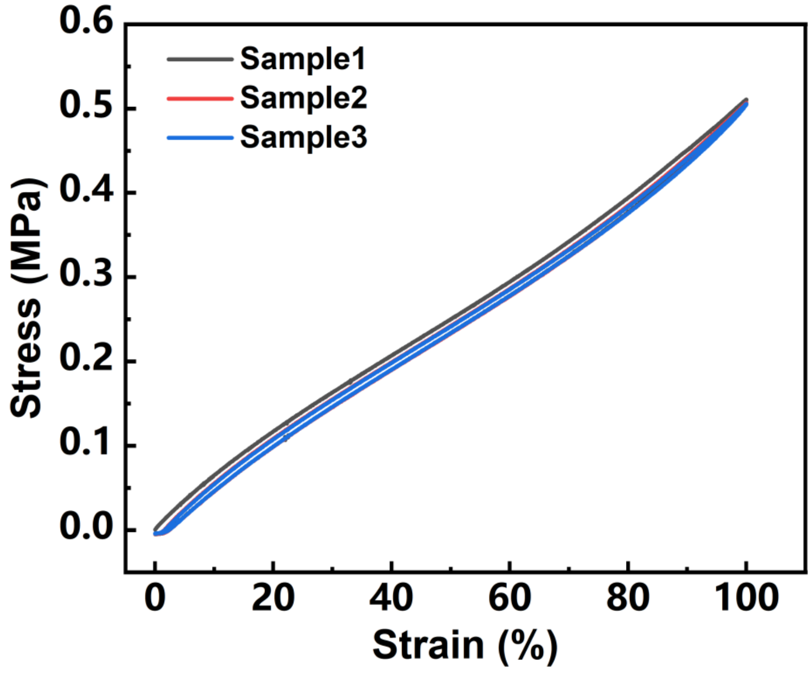

Figure S1. Experimental results for the tensile properties of the sample pieces (length: 2 cm, width: 1 cm, thickness: 1 mm).

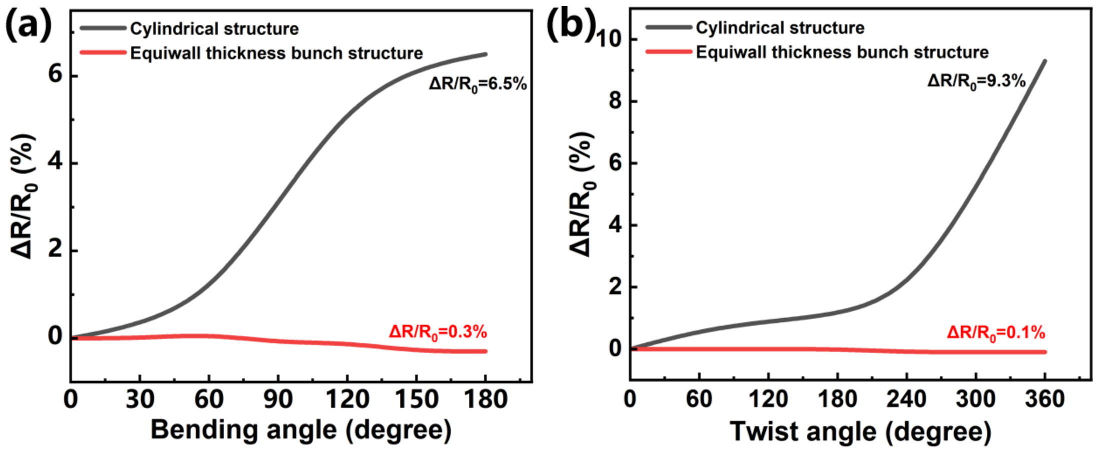

Figure S2. (a) The resistance changes of bunch structure and cylindrical structure during bending are compared. (b) The resistance changes of bunch structure and cylindrical structure during twist are compared.

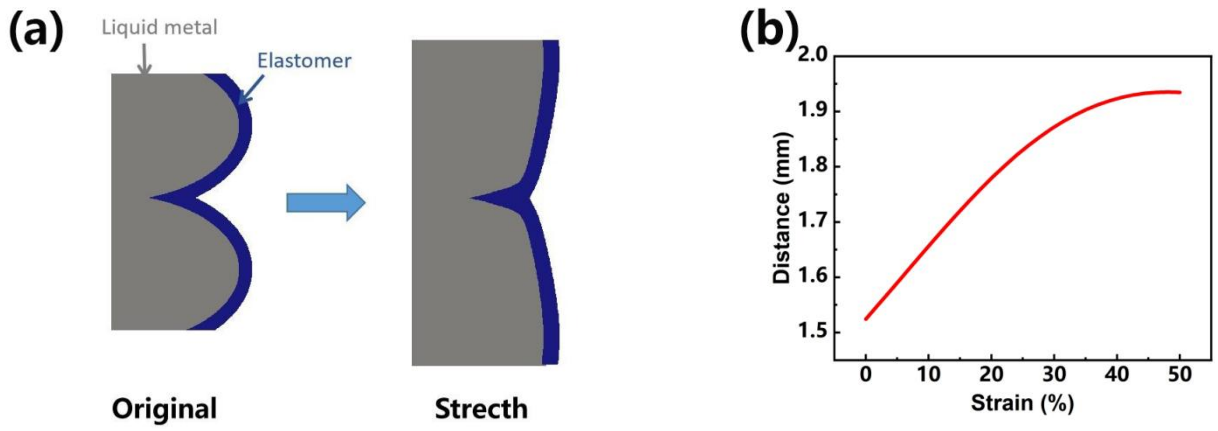

Figure S3. (a) Simulation diagram of structural change of stretchable conductor with equiwall thickness linear bunch conductive network during stretching. (b) Radius change at neck.

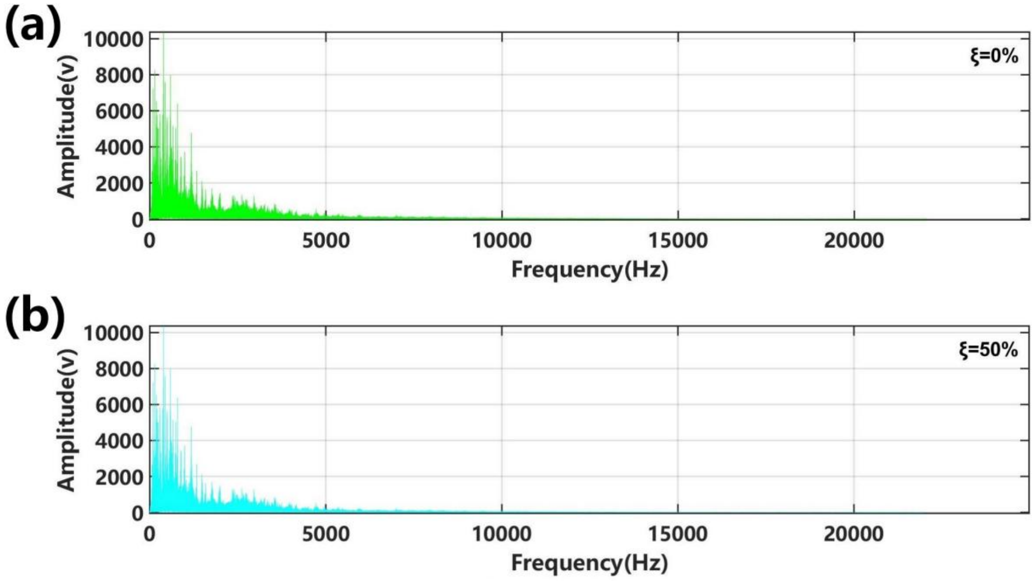

Figure S4. (a) Spectrum of initial state voltage waveform. (b) Spectrogram of voltage waveform at 50% tensile stress.

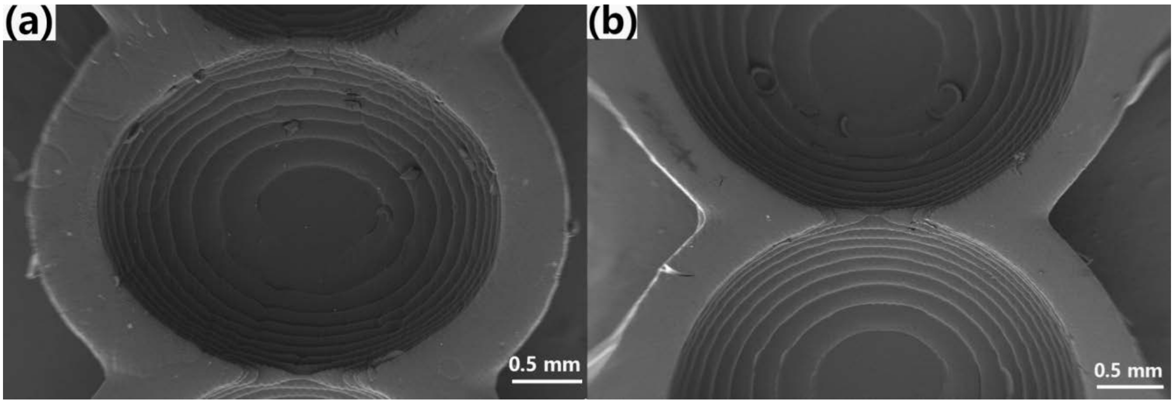

Figure S5. Cross-sectional section diagram of equiwall thickness bunch structure.
